# Impact of a “Brain Protection Bundle” in Reducing Severe Intraventricular Hemorrhage in Preterm Infants <30 Weeks GA: A Retrospective Single Centre Study

**DOI:** 10.3390/children8110983

**Published:** 2021-10-31

**Authors:** Nishkal Persad, Edmond Kelly, Nely Amaral, Angela Neish, Courtney Cheng, Chun-Po Steve Fan, Kyle Runeckles, Vibhuti Shah

**Affiliations:** 1 Department of Paediatrics, Mount Sinai Hospital, 600 University Avenue, Toronto, ON M5G 1X5, Canada; nishkal10@yahoo.com (N.P.); Edmond.Kelly@sinaihealth.ca (E.K.); chengcourtney1@gmail.com (C.C.); 2 Department of Nursing, Mount Sinai Hospital, 600 University Avenue, Toronto, ON M5G 1X5, Canada; Nely.Amaral@sinaihealth.ca (N.A.); Angela.Neish@sinaihealth.ca (A.N.); 3 Rogers Computational Program, Ted Rogers Centre for Heart Research, Peter Munk Cardiac Centre, University Health Network, 585 University Avenue, 2RFE-417, Toronto, ON M5G 2N2, Canada; S.Fan@uhnresearch.ca (C-P.S.F.); Kyle.Runeckles@uhnresearch.ca (K.R.)

**Keywords:** preterm infant, intraventricular hemorrhage, brain injury

## Abstract

Background: despite advances in perinatal care, periventricular/intraventricular hemorrhage (IVH) continues to remain high in neonatal intensive care units (NICUs) worldwide. Studies have demonstrated the benefits of implementing interventions during the antenatal period, stabilization after birth (golden hour management) and postnatally in the first 72 h to reduce the incidence of IVH. Objective: to compare the incidence of severe intraventricular hemorrhage (IVH ≥ Grade III) before and after implementation of a “brain protection bundle” in preterm infants <30 weeks GA. Study design: a pre- and post-implementation retrospective cohort study to compare the incidence of severe IVH following execution of a “brain protection bundle for the first 72 h from 2015 to 2018. Demographics, management practices at birth and in the NICU, cranial ultrasound results and short-term morbidities were compared. Results: a total of 189 and 215 infants were included in the pre- and post-implementation phase, respectively. No difference in the incidence of severe IVH (6.9% vs. 9.8%, *p* = 0.37) was observed on the first cranial scan performed after 72 h of age. Conclusion: the implementation of a “brain protection bundle” was not effective in reducing the incidence of severe IVH within the first 72 h of life in our centre.

## 1. Introduction

Perinatal-neonatal medicine has evolved over time, with tremendous improvement in the survival rates of preterm infants born <30 weeks gestational age (GA) [1]. With increased survival at the limits of viability, there is concern regarding the potential increase in both short-term neonatal morbidities and long-term adverse neurodevelopmental outcomes [2]. In a recent study published by Synnes et al. [3] including data for all infants born <29 weeks GA across 28 Canadian Neonatal Network (CNN) sites from 2009–2011, the authors showed that 46% of all survivors had some level of neurodevelopmental impairment at 18–21 months, with brain injury being the highest risk factor for adverse outcome.

Despite advances in perinatal care, periventricular/intraventricular hemorrhage (IVH) is one short-term morbidity which continues to be remain high in neonatal intensive care units (NICUs) worldwide [4,5]. The immaturity of the developing brain of preterm infants increases their vulnerability to injury post-delivery. Severe IVH, defined as ≥ Grade III according to Papile’s classification [6] is strongly associated with adverse long-term neurodevelopmental outcomes particularly in the realms of motor and cognitive development [3,7]. According to the data from CNN, the rate of severe IVH has remained unchanged for the 5-year period from 2013 to 2017 across Canada despite many quality improvement initiatives, with the incidence being 20–22% in infants at 22–25 [6,7] and 7–10% in those at 26–28 [6,7] weeks GA, respectively [8].

To-date, there have been several interventions with proven benefits in the reduction of severe IVH, including antenatal corticosteroids [9,10] and prophylactic indomethacin [11,12]. The concept of the “golden hour” with the aim of optimizing management in the perinatal and immediate post delivery period has been extensively reviewed [13,14,15]. Similarly, the midline head positioning and minimal handling for the first few days of life has been investigated with variable results in preventing IVH [16,17,18]. The avoidance of hemodynamic fluctuation and optimized ventilation strategies have shown to be effective neuroprotection interventions with good quality of evidence [19,20,21]. Further, there are observational studies and quality improvement projects that utilize many of these core concepts that have been incorporated in “bundle of care” for reducing IVH in preterm infants [22,23,24,25].

In 2015, the incidence of severe IVH in infants <30 weeks GA at our centre was reported to be 11% which is much higher than the benchmark rate reported by CNN. This led to the creation and implementation of a “brain protection bundle” which included interventions beginning from the antenatal period until the first 72 h, with the goal to reduce the incidence of severe IVH in this population. The aim of this study is to evaluate the effectiveness of a “brain protection bundle” in reducing the incidence of severe IVH for preterm infants <30 weeks GA.

## 2. Methods

### 2.1. Setting and Study Design

This was a retrospective cohort study conducted at Mount Sinai Hospital (MSH), Toronto with a pre- and post-implementation design. The two time periods were as follows: (1) pre-implementation phase (1 November 2015 to 31 October 2016) and (2) post-implementation phase (1 March 2017 to 28 February 2018) when the “brain protection bundle” was implemented. The time between 1 December 2016 and 29 February 2017 was considered a “washout period” as the team was integrating the bundle in clinical practice.

### 2.2. Inclusion and Exclusion Criteria

All inborn preterm infants <30 weeks GA during the 2 time periods were included. Infants who were outborn, offered palliative care at birth, had major congenital anomalies, died or were transferred to another hospital prior to a cranial ultrasound were excluded.

### 2.3. Brain Protection Bundle Development Process

In the first 6 months of 2016 the MSH Evidence-Based Practice for Improving Quality (EPIQ) IVH working group was established and consisted of healthcare professionals (physicians, nurses and respiratory therapists) with the goal of reducing severe IVH by 30% within 12 months. Evidence-based bundle of practices were reviewed, and a modified version of the “brain protection bundle” was developed, specifically to accommodate family integrated care within the confines of the physical and working environment of the single room design NICU at MSH.

Between October to November 2016, each element of the bundle was critiqued by the IVH working group and brought up at staff neonatologists’ weekly meeting for an update and discussion before deciding to trial the bundle in the NICU. This joint collaboration was very important as the bundle not only affected physicians’ and respiratory therapists’ care, but also played a significant role in the day-to-day nursing care. One particular area of concern about the bundle was in regards to family interaction with their infants, particularly skin-to-skin contact, as this was seemingly contraindicated given the mandate for minimum handling during the first 72 h of life. Other challenges included establishing and maintaining an environment with minimal noise disturbances and preventing hypothermia during transport to the NICU from the resuscitation room post-delivery. In this case, 12 different educational handouts were created between October to November 2016, which aimed to support nurses, NICU physicians and other allied healthcare professionals as well as families in the changes to practice with any infant < 30 weeks GA. Families were encouraged to perform hand hugging (or therapeutic touch) with their infants once deemed feasible.

In essence, the interventions bundle started with optimization of antenatal care, ensuring administration of antenatal steroids, identifying mothers with suspected or confirmed chorioamnionitis, and determining any other risk factors that may cause hemodynamic instability in the preterm infant. It continued in the peripartum and immediate post-delivery period with provision of the “brain protection bundle package”, debriefing the team prior to the delivery, preparing the warmer for the infant, encouraging delayed cord clamping (DCC) and resuscitation and stabilization in keeping with the protocol for the extremely low birth weight infant. Once the infant was stabilized, bundle measures continued for the first 72 h by placing a card on the door of the infant’s room with the date and time of birth and date and time when the measures were to be lifted, ensuring minimal noise, keeping the head of the bed elevated to ~30 degrees, keeping the head and body in midline, minimizing handling by grouping all assessments completed with 2-person handling and ensuring adequate pain and stress management (Supplementary Table S1).

Nursing and staff leads identified an appropriate time (last week of October 2016) to “trial run” the bundle on 3 infants of 23, 27 and 29 weeks GA. These GAs were targeted to ensure that the components of the bundle could be applied to a range of GAs and troubleshoot if any problems were identified at the bedside. Minor modifications were made to the “brain protection bundle” based on this “trial run” and were implemented as a practice change in January 2017.

### 2.4. Clinical Data and Variables

Data were retrieved from the electronic medical record and CNN database using a pre-designed data collection form for this study. Data were collected on the following variables from time of birth until transfer to the NICU: maternal characteristics including antenatal corticosteroids and magnesium sulfate administration, prolonged rupture of membranes (>18 h), maternal chorioamnionitis and treatment with antibiotics, placental abruption and mode of delivery; neonatal demographics including GA at birth, birth weight, sex, small for GA (<10th%tile), Apgar scores at 1 and 5 min of age, Score for Neonatal Acute Physiology-II (SNAP II) score [26,27]; and neonatal management characteristics at birth including DCC for >30 s, trial of continuous positive pressure ventilation (CPAP) at birth, intubation within the first hour of birth, premedication for intubation, surfactant administration, and need for cardiopulmonary resuscitation (CPR) including administration of epinephrine and fluid resuscitation (use of normal saline and/or packed red blood cell [PRBC] transfusion).

In addition, data on surfactant administration, blood gas parameters (pH and partial pressure of carbon dioxide [pCO_2_]), need for inotropes and CPR (including epinephrine administration, bicarbonate infusion, fluid resuscitation including PRBC transfusion), echocardiography and need for medical treatment for patent ductus arteriosus (PDA), hypoglycemia (blood glucose level < 2.6 mmol/L), hypothermia (<36 °C) and whether lumbar puncture was performed during the first 72 h of life were collected.

Prior to implementation of the “brain protection bundle”, cranial ultrasound scans (CUS) were performed at the discretion of the staff neonatologist depending on the clinical status of the infant at birth and course in the first few days of life. As part of the “brain protection bundle”, CUS was performed after 72 h of birth unless the infant was critically ill and the results could potentially influence direction of care. Results of the first CUS at ≥72 h of life, CUS with the worst finding during NICU stay based on Papile’s classification^6^ and information on short-term morbidities including late onset sepsis (blood culture or cerebrospinal fluid positive beyond 48 h of life), bronchopulmonary dysplasia (BPD, defined as the need for oxygen or respiratory support at ≥ 36 weeks) [28], necrotizing enterocolitis (NEC ≥ stage 2, using the modified Bell’s staging criteria) [29], severe retinopathy of prematurity (ROP, defined as ≥ stage 3) [30] and mortality after 72 h of life were collected.

### 2.5. Statistical Analysis

Data were stored on a Microsoft Office Excel Database designed for the study. Clinical characteristics were summarized by means and standard deviation using *t*-test or medians and interquartile range (IQR) for continuous variables using Wilcoxon rank-sum test, while frequencies and proportions were reported for dichotomous and polytomous variables. For dichotomous/polytomous variables, differences were assessed with Fisher’s exact tests. Statistical significance was achieved at a *p*-value < 0.05. Statistical analysis was generated using SAS software, Version 9.4 of the SAS System for Windows^®^ (copyright 2016, SAS Institute Inc., Cary, NC, USA).

### 2.6. Research Ethics Approval

The study was approved by the local Research Ethics Board.

## 3. Results

**Figure 1:**
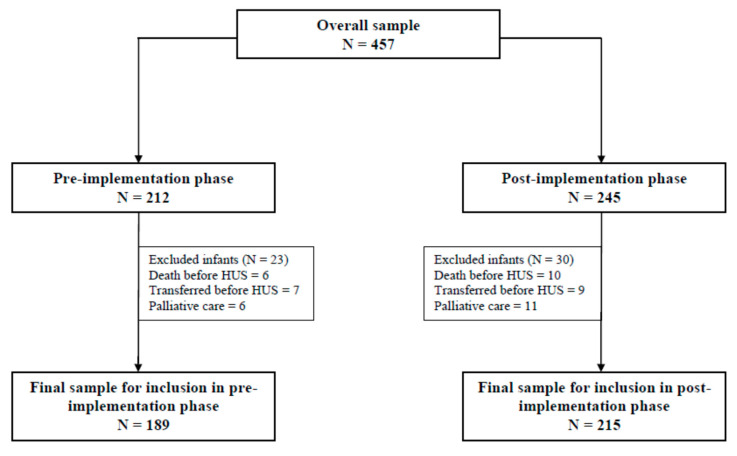
Study population.

Of the 457 eligible infants over the 2 time periods, a total of 404 infants were included in the study, 189 infants in the pre-implementation phase and 215 infants in the post-implementation phase (Figure 1).

Maternal and neonatal demographic characteristics and neonatal management characteristics at birth are presented in Table 1. 

Despite a statistically significant lower rate of maternal chorioamnionitis in the post-implementation phase, a higher proportion of mothers received antibiotics prior to delivery (*p* < 0.001). Similarly, GA, birth weight and SNAP-II score > 20 were statistically significantly lower while multiple births were higher in the post-implementation phase (*p* < 0.05 for all). There was no difference in the neonatal management characteristics between the 2 time periods (*p* values were > 0.05 for all).

The neonatal characteristics for the first 72 h of life are summarized in Table 2.

There were no statistically significant differences for all variables except for the rate of blood transfusions (10.6% vs. 20.5%, *p* = 0.009) and prophylactic indomethacin administration (15.9% vs. 29.8%, *p* < 0.001), while an increased trend on the use of sodium bicarbonate infusions (5.8% vs. 11.6%, *p* = 0.053) was noted in the post-implementation phase. The rate of CUS performed within the first 72 h of life was significantly reduced in the post-implementation phase (49.2% vs.14.4%, *p* < 0.001). Short-term neonatal outcomes are summarized in Table 3.

There was no statistically significant difference in the incidence of severe IVH between the pre- and post-implementation period (6.9% vs. 9.8%, *p* = 0.37) based on the first cranial ultrasound scan performed after 72 h of life. Similarly, the composite outcome of any ≥ Grade 3 IVH /PVL/hydrocephalus during the entire NICU stay was not different between the groups (11.6% vs. 15.3%, *p* = 0.31). The incidence of mortality and short-term neonatal morbidities were similar for both groups. The study population was further stratified based on GA of < or ≥26 weeks and found that the incidence of severe IVH was similar (Table 4).

## 4. Discussion

In this study we were unable to provide compelling evidence to suggest that the implementation of a “brain protection bundle” was effective in reducing the incidence of severe IVH in our population even after stratifying for GA. The post-implementation data was collected after a wash-out period to account for the adoption of the IVH bundle by the healthcare professionals in clinical practice.

Potential explanations for our negative findings include infants in the post-implementation phase were of lower GA and BW and these infants are at the highest risk of IVH due to the fact that 95% of these infants demonstrate pressure passive cerebral blood flow at 20–50% of the time [31]; and a higher incidence of multiple gestation, which in itself is an independent risk factor for IVH [32]. Both of these are non-modifiable factors. In addition, infants in the post-implementation had higher rates of packed red blood cell transfusion. The need for transfusion may be reflective of a sicker, more hemodynamically unstable infant and additionally, blood transfusion in itself is an independent risk for intracranial bleeding during this period [33]. As well, there was a trend towards increased utilization of bicarbonate infusions within the first 72 h of life. The use of sodium bicarbonate administration particularly in preterm infants, is a known risk factor for IVH possibly due to its hypertonicity coupled with rapid infusion as a bolus [34,35].

There were no statistically significant differences in other factors that have been implicated in the occurrence of IVH, including ventilatory management, abnormal pCO_2_ (<35 mm·Hg and/or >55 mm·Hg) [36,37] early use of volume expanders and inotropes, [38] and occurrence of hypothermia [39]. Elevated pCO_2_ levels > 60 mm Hg are associated with impaired cerebral auto-regulation and vasodilatation which increases exponentially with increasing pCO_2_ levels [37]. Even though there was no statistically significant difference in the administration of complete course of antenatal steroids between the two time periods, the rate was lower in the post-implementation phase (75.7%) as compared to the pre-implementation phase (81.5%). The reason for this discrepancy is not clear, however it may be that women may have presented in preterm labor and there was insufficient time to provide a complete course of steroids. Further, we did not collect information on whether there was a difference in the percentage of women who received a single dose of steroids or none between the two time periods. Lastly, the negative findings may be due to lack of power in the study as there was no a priori sample size calculation.

Following the implementation of the “brain protection bundle”, there was a statistically significant reduction in the frequency of CUS performed in the first 72 h of life from 49.2% vs.14.4%, *p* < 0.001which is an indirect reflection that healthcare professionals were following the care bundle practices. The goal of performing CUS after the first 72 h was to minimize handling and stress to these vulnerable infants during this very sensitive period, which may in fact be neuroprotective. Even though there was a reduction in CUS being performed, the rate of lumbar punctures did not change between the 2 phases of the study and is an independent risk factor for severe IVH [40].

Our findings are in keeping with the study by Gross et al. [41] who evaluated the effectiveness of a neonatal care bundle similar to our “brain protection bundle” to reduce the rate of IVH and failed to demonstrate any difference in the overall and severe grades of IVH. However, they are in contrast to several other quality improvement (QI) studies that have targeted initiatives to reduce IVH or short-term neonatal outcomes with varying degrees of success [22,23,24,25] Schmid et al. demonstrated that the introduction and prospective monitoring of bundle of care practices developed on the basis of risk factors reduced the overall rate of IVH (from 22.1 to 10.5%) and severe IVH (from 9.1 to 3.7%) over a period of 23 months. Their success was attributed to the inclusion of teamwork between disciplines and professions, evidence-based identification of risk factors, visit and learning from a site with lower IVH incidence, development of site-specific bundle and weekly meetings to check for adherence to the bundle and case-based discussion. However, the results should be interpreted with cautions as the study period (31 vs. 23 months) and sample size (265 vs. 191 infants) differed between the two time periods. Furthermore, the authors state that the centre-specific measures can be translated to other sites to a limited extent. Similarly, Ellsbury et al. [23] using the Kotter organizational change model to improve care in multiple domains was successful in reducing mortality, NEC, ROP and late-onset sepsis while BPD and severe IVH showed slight improvement or remained stable. Lapcharoensap et al. [24] used a pre-post-implementation study design, applied delivery room management interventions through Collaborative QI and compared outcomes with a single–site QI model and a non-participant site. A significant reduction in the outcome of BPD and composite outcome of BPD and death was noted in the Collaborative QI group. Furthermore, there was a reduction in overall and severe IVH, severe ROP and composite outcome of death and severe IVH or death and severe ROP both in the Collaborative QI and non-participant groups. The authors concluded that institutions committed to improving delivery room practices can impact outcome. In a recent study, Chiriboga et al. [25] showed that through a sustained multi-disciplinary QI initiative involving the introduction of an IVH protection bundle, there was a reduction in the baseline rate of severe IVH of 24 to 9.7% over a 4-year period.

The pathogenesis of IVH is multi-factorial but at its core, there are two fundamental factors: the intrinsic fragility of the germinal matrix and the inability of preterm infants to auto-regulate and control fluctuations in their cerebral blood flow, especially within the first 72 h of life. Our bundle encompassed antenatal, delivery room and postnatal practices including delayed cord clamping, respiratory management including the use of non-invasive ventilation and management of pCO_2_ and acid-base balance to avoid fluctuation in cerebral blood flow.

Major limitations of our study include inclusion of multiple interventions within the bundle, no prospective monitoring of adherence/compliance to the various components of the bundle, heterogeneity in implementing the components of the bundle at an individual level of the healthcare team, potential variation in reporting of CUS as they were reported by several radiologists, and the short-duration of outcome assessment for IVH (1-year period). Further, we only reported outcomes on infants while they were in the NICU at our institution and did not capture information on whether there was progression or worsening with regards to ventricular dilatation or occurrence of other short-term neonatal morbidities after transfer to a level II NICU.

Several well-known quality improvement initiatives have been established worldwide including CNN-EPIQ [42], CPQCC [43], and VON-NIC/Q [44] which have shown improvements in neonatal outcomes. However, there are also large-scale projects such as VON’s Reduce Lung Injury [45] and the NICHD-NRN on BPD [46] which showed no improvement in outcomes despite implementation of potentially best practices. It is increasingly recognized that regardless of the best intentions many QI projects do not achieve their goals and challenges are multi-faceted. In spite of having the data on the rate of severe IVH and its consequences, we were not able to make a change. It is important to remember that reduction in the rate of IVH in other centres does not necessarily guarantee the benefits in other centres. Potential barriers to improvement include convincing healthcare teams that there is a problem (“buy-in”), over-ambitious goals in implementing the different components of the bundle, not recognizing the demands placed on the front-line staff and the support they received, lack of perceived ownership and differences in opinions between disciplines (e.g., physicians, nurses). Further, over time the healthcare team may become indifferent in their adherence to the components of the bundle and may need reinforcement for sustainability of the bundle in clinical practice [47]. To-date, there have been no randomized controlled trials evaluating the effectiveness of brain protection bundles to reduce the incidence of IVH. Most observational studies are hypothesis generating and there is a need for randomized controlled trial to confirm or refute the benefits of this bundle on IVH as an outcome.

## 5. Conclusions

Even though, there was no reduction in severe IVH in preterm infants <30 weeks GA, the components of the bundle were relevant clinically and provided opportunities for our healthcare team to deliver optimal care while minimizing unnecessary handling and for families to provide essential skin to skin contact using hand hugging. Ongoing renewed efforts will be continued to modify the bundle, prospectively monitor the implementation of interventions, case- analysis and culture change to reduce severe IVH in our centre.

## Figures and Tables

**Table 1:** Maternal and neonatal demographics and management characteristics.

Variable ^1^	Overall (N = 404)	Pre-Implementation Phase (N = 189)	Post-Implementation Phase (N = 215)	*p* Value ^2^
**Maternal characteristics**				
Complete course of antenatal steroids	316 (78.4%)	154 (81.5%)	162 (75.7%)	0.27
Rupture of membranes > 18 h	139 (34.4%)	70 (37.0%)	69 (32.1%)	0.34
Maternal chorioamnionitis ^3^	323 (81.0%)	161 (85.6%)	162 (76.8%)	0.03
Maternal antibiotics prior to delivery	346 (85.6%)	140 (74.1%)	206 (95.8%)	<0.001
Placental abruption	32 (7.9%)	17 (9.0%)	15 (7.0%)	0.47
Intrapartum magnesium sulphate administration	370 (91.6%)	172 (91.0%)	198 (92.1%)	0.72
Vaginal delivery	185 (45.8%)	88 (46.6%)	97 (45.1%)	0.84
**Neonatal characteristics**				
Gestational age at birth (weeks)	27.0 ± 1.9	27.3 ± 1.7	26.8 ± 2.1	0.01
Birth weight (grams)	956 ± 282	1005 ± 285	914 ± 274	0.001
Small for gestational age (<10th%tile)	35 (8.7%)	14 (7.4%)	21 (9.8%)	0.48
Sex (% Male)	224 (55.4%)	114 (60.3%)	110 (51.2%)	0.07
Multiple births	115 (28.5%)	48 (25.6%)	67 (31.1%)	0.02
Apgar score at 1 min	6 (2–8)	6 (2–8)	6 (2–8)	0.72
Apgar score at 5 min	8 (6–9)	8 (6–9)	8 (5–9)	0.20
SNAP II score > 20	109 (27.0%)	63 (33.3%)	46 (21.4%)	0.01
Umbilical artery cord pH < 7.0 ^4^	18 (5.0%)	12 (7.2%)	6 (3.1%)	0.09
**Neonatal management characteristics at birth**				
Delayed cord clamping > 30 s	232 (57.4%)	99 (52.4%)	133 (61.9%)	0.06
Trial of CPAP at resuscitation *	315 (78.0%)	155 (82.0%)	160 (74.4%)	0.07
Intubation within first hour of life	169 (41.8%)	75 (39.7%)	94 (43.7%)	0.42
Premedication used for first intubation attempt ^5^	1 (0.2%)	0 (0.0%)	1 (0.5%)	1.00
CPR given at delivery *	12 (3.0%)	9 (4.8%)	3 (1.4%)	0.08
Epinephrine needed after delivery	6 (1.5%)	4 (2.1%)	2 (0.9%)	0.42
Surfactant administration within first hour of life	138 (34.2%)	56 (29.6%)	82 (38.1%)	0.08
Fluid bolus administration within first hour of life ^6^	12 (3.0%)	9 (4.8%)	3 (1.4%)	0.08

^1^ Results are presented as mean, standard deviation; median, inter-quartile range and number, percentage as appropriate. ^2^ Significance was assessed by Wilcoxon rank-sum tests and Student’s t-test for continuous variables and Fisher’s exact tests for differences between dichotomous/polytomous variables. ^3^ Data available for 399 patients (188 pre- and 211 post-implementation). ^4^ Data available for 360 patients (166 pre- and 194 post-implementation). ^5^ Data available for 403 patients (188 pre- and 215 post-implementation). ^6^ Fluid bolus (≥ 10 mL/kg of normal saline or blood transfusion). * CPAP = Continuous positive airway pressure; CPR = Cardiopulmonary resuscitation.

**Table 2:** Neonatal management characteristics for the first 72 h of life.

Variable ^1^	Overall (N = 404)	Pre-Implementation Phase (N = 189)	Post-Implementation Phase (N = 215)	*p* Value ^2^
Endotracheal intubation	114 (28.2%)	51 (27.0%)	63 (29.3%)	0.66
Premedication for intubation ^3^	102 (89.4%)	43 (84.3%)	59 (93.6%)	0.30
Surfactant administration	148 (36.6%)	61 (32.3%)	87 (40.5%)	0.10
Abnormal pCO_2_ (<35 or ≥55 mmHg)	286 (70.8%)	137 (72.5%)	149 (69.3%)	0.44
Abnormal pH (<7.2 or ≥7.4)	214 (53%)	96 (50.8%)	118 (54.9%)	0.36
Need for inotropes	21 (5.2%)	10 (5.3%)	11 (5.1%)	1.00
Need for cardiopulmonary resuscitation	3 (0.7%)	2 (1.1%)	1 (0.5%)	0.60
Epinephrine administration for resuscitation	2 (0.5%)	2 (1.1%)	0 (0.0%)	0.22
Bicarbonate infusion administration	36 (8.9%)	11 (5.8%)	25 (11.6%)	0.053
Fluid bolus (normal saline ≥ 10 mL/kg)	54 (13.4%)	25 (13.2%)	29 (13.5%)	1.00
Packed red blood cell transfusion	64 (15.8%)	20 (10.6%)	44 (20.5%)	0.009
Prophylactic indomethacin	94 (23.3%)	30 (15.9%)	64 (29.8%)	<0.001
Need for echocardiography ^4^	78 (19.4%)	33 (17.5%)	45 (21.0%)	0.38
Hemodynamically significant patent ductus arteriosus	28 (6.9%)	14 (7.4%)	14 (6.5%)	0.84
Medical treatment for patent ductus arteriosus	18 (4.5%)	10 (5.3%)	8 (3.7%)	0.48
Blood glucose level (≤2.6 mmol/L)	131 (32.4%)	61 (32.3%)	70 (32.6%)	1.00
Hypothermia (<36 °C)	47 (11.6%)	16 (8.5%)	31 (14.4%)	0.086
Early-onset sepsis	14 (3.5%)	5 (2.6%)	9 (4.2%)	0.43
Lumbar puncture performed	18 (4.5%)	9 (4.8%)	9 (4.2%)	0.81
Platelet count < 100 × 10^9^/L	58 (14.4%)	29 (15.3%)	29 (13.5%)	0.67
First cranial ultrasound (<72 h of life)	124 (30.7%)	93 (49.2%)	31 (14.4%)	<0.001

^1^ Results are presented as number, percentage. ^2^ Significance was assessed by Wilcoxon rank-sum tests and Student’s *t*-test for continuous variables and Fisher’s exact tests for differences between dichotomous/polytomous variables. ^3^ Data available for 402 patients (189 pre- and 213 post-implementation). ^4^ Data available for 403 patients (188 pre- and 214 post-implementation).

**Table 3:** Short-term neonatal outcomes.

Variable ^1^	Overall (N = 404)	Pre-Implementation Phase (N = 189)	Post-Implementation Phase (N = 215)	*p* Value ^2^
**First Cranial ultrasound scan (≥72 h of life)**				0.63
No intraventricular hemorrhage (IVH)	242 (59.9%)	116 (61.4%)	126 (58.6%)	
Grade I IVH	90 (22.3%)	45 (23.8%)	45 (20.9%)	
Grade II IVH	38 (9.4%)	15 (7.9%)	23 (10.7%)	
Grade III IVH	6 (1.5%)	2 (1.1%)	4 (1.9%)	
Grade IV IVH	27 (6.7%)	11 (5.8%)	16 (7.4%)	
Periventricular leukomalacia (PVL)	1 (0.2%)	0 (0.0%)	1 (0.5%)	
≥Grade III IVH/PVL (on first scan ≥ 72 h of life)	34 (8.4%)	13 (6.9%)	21 (9.8%)	0.37
**Worst cranial ultrasound scan result anytime during NICU hospitalization**				0.25
No IVH	194 (48.0%)	98 (51.9%)	96 (44.7%)	
Grade I IVH	115 (28.5%)	50 (26.5%)	65 (30.2%)	
Grade II IVH	40 (9.9%)	19 (10.1%)	21 (9.8%)	
Grade III IVH	8 (2.0%)	3 (1.6%)	5 (2.3%)	
Grade IV IVH	31 (7.7%)	14 (7.4%)	17 (7.9%)	
PVL	10 (2.5%)	2 (1.1%)	8 (3.7%)	
Hydrocephalus requiring intervention ^3^	6 (1.5%)	3 (1.6%)	3 (1.4%)	
≥Grade III IVH/PVL/Hydrocephalus	55 (13.6%)	22 (11.6%)	33 (15.3%)	0.31
**Major neonatal morbidities ^4^**				
Bronchopulmonary dysplasia	93 (23.0%)	41 (21.7%)	52 (24.2%)	0.83
Necrotizing enterocolitis ≥ Stage 2	52 (12.9%)	22 (11.6%)	30 (14%)	0.88
Late-onset sepsis	86 (21.3%)	30 (15.9%)	56 (26.0%)	0.02
Retinopathy of prematurity > Stage 3	24 (5.9%)	10 (5.3%)	14 (6.5%)	0.81
Mortality (≤72 h of life after completion of cranial ultrasound)	2 (0.5%)	2 (1.1%)	0 (0.0%)	0.22
Mortality (>72 h of life)	31 (7.7%)	13 (6.9%)	18 (8.4%)	0.58

^1^ Results are presented as number, percentage. ^2^ Significance was assessed by Wilcoxon rank-sum tests and Student’s *t*-test for continuous variables and Fisher’s exact tests for differences between dichotomous/polytomous variables. ^3^ Interventions include therapeutic lumbar punctures, Ommaya reservoir or ventriculoperitoneal shunt insertion. ^4^ Diagnosis made in the NICU prior to death or transfer to another institution.

**Table 4:** Incidence of ≥ grade III intraventricular hemorrhage (IVH) based on gestational age.

Variable ^1^	Overall (N = 404)	Pre-Implementation Phase (N = 189)	Post-Implementation Phase (N = 215)	*p* Value ^2^
**Infants < 26 weeks gestational age**	**115**	**40**	**75**	
Gestational age (weeks)	24.5 ± 0.9	24.8 ± 0.9	24.4 ± 0.9	0.07
Birth weight (grams)	682 ± 127	696 ± 134	674 ± 123	0.40
Composite ≥ Grade III IVH/periventricular leukomalacia (PVL) at first scan ≥ 72 h	17 (15%)	5 (13%)	12 (16%)	0.78
Composite ≥ Grade III IVH/PVL/Hydrocephalus anytime during hospitalization	31 (27%)	8 (20%)	23 (31%)	0.27
**Infants 26–30 weeks gestational age**	**289**	**149**	**140**	
Gestational age (weeks)	28.0 ± 1.2	28.0 ± 1.2	28.1 ± 1.2	0.73
Birth weight (grams)	1066 ± 251	1088 ± 256	1042 ± 245	0.12
Composite ≥ Grade III IVH / PVL at first scan ≥ 72 h	17 (5.9%)	8 (5.4%)	9 (6.4%)	0.80
Composite ≥ Grade III IVH / PVL/Hydrocephalus anytime during hospitalization	24 (8.3%)	14 (9.4%)	10 (7.1%)	0.53

^1^ Results are presented as mean, standard deviation and number, percentage as appropriate. ^2^ Significance was assessed by Wilcoxon rank-sum tests and Student’s *t*-test for continuous variables and Fisher’s exact tests for differences between dichotomous/polytomous variables.

## Data Availability

The data presented in this manuscript are available on request from the corresponding author.

## Supplementary Materials

The following are available online at https://www.mdpi.com/article/10.3390/children8110983/s1, Table S1: Components of the brain protection bundle.

## Abbreviations

BPD—Bronchopulmonary dysplasia; CNN—Canadian Neonatal Network; CNN-EPIQ—Canadian Neonatal Network Evidence-Based Practice for Improving Quality; CPAP—Continuous positive airway pressure; CPQCC—California Perinatal Quality Care Collaborative; CPR—Cardiopulmonary resuscitation; CUS—Cranial ultrasound; DCC—Delayed cord clamping; ECHO—Echocardiogram; GA—Gestational age; IVH—Intraventricular hemorrhage; LP—Lumbar puncture; MgSO_4_—Magnesium sulfate; MSH—Mount Sinai Hospital; NEC—Necrotizing enterocolitis; NICHD-NRN—National Institute of Child Health and Human Development-Neonatal Research Network; NICU—Neonatal intensive care unit; pCO_2_—Partial pressure of carbon dioxide; PDA—Patent ductus arteriosus; PVL—Periventricular leukomalacia; PROM—Prolonged rupture of membranes; PRBC—Packed red blood cell; RDS—Respiratory distress syndrome; ROP—Retinopathy of prematurity; SNAP—Score for Neonatal Acute Physiology; VON—Vermont Oxford Network; VON-NIC/Q—Vermont Oxford Network Newborn Improvement Collaborative for Quality.
