# Supplementary material for: Impact of a “Brain Protection Bundle” in Reducing Severe Intraventricular Hemorrhage in Preterm Infants <30 Weeks GA: A Retrospective Single Centre Study
Source: Children (Basel). 2021 Oct 31;8(11):983. doi: 10.3390/children8110983 (PMC8624779; doi:10.3390/children8110983)
Supplement: Supplementary file 1 [file children-08-00983-s001.zip › children-1395758-SI.pdf]

"Promoting Brain Health"
NICU IVH Bundle
For all Infants less than 30 Weeks Gestational Age
IVH Bundle for the first 72 h in the NICU
**** All interventions are "as medically indicated"****

Maternal interventions

- Optimization of antenatal steroid administration
- Identifying mothers with suspected or confirmed chorioamnionitis
- Identifying any other risk factors that may lead to ehmodynamic instability in infants

Identify Infant

- Fill out IVH ID card with time of birth and 72 h END time clearly written
- Place IVH cards on incubator and on door

Head of the bed should be elevated

- **Elevate head of bed** – At the highest level so that when you pull out X-ray tray it slides out easily
- If bed needs to be levelled-slow and steady
- "Nesting" of infant to keep from sliding down

Keep head and body midline (ears, shoulder and hips should be in a straight line)

- Use rolls or "froggie" whenever possible
- No prone position for 72 h. No lateral positioning (unless clinically indicated)
- **Two person handling and repositioning for:**
  - Any repositioning where there is a risk of turning head
  - X-ray : Avoid levelling the bed if possible—consider dorsal-decubitus (cross-table) view
  - Weight or linen change (if indicated)
  - Transfer (if indicated)
  - Change/removal of polyethylene occlusive plastic wrap up to 6 h (as per policy)
  - Skin to skin if permitted
  - Back assessment—rotate laterally once daily unless clinically indicated

Medications

- Premedication preferred for all endotracheal tube insertions (unless in emergency)
- Gentle application of eye ointment (if not already given in Resuscitation room)
- **Mepitel** dressings—for all infants $22^{0/7}$ to $24^{6/7}$ weeks gestation—use Mepitel dressing over chest to secure leads (avoid direct contact of leads to skin)

BP Monitoring

- **When UAC present**
  - Do not use manual BP unless clinically unstable and correlation is required (inotropes)
  - Any flushing/withdrawal needs to be slow and steady (minimum 30 s each step)
- **When no UAC present**
  - *Greater than 27 weeks* - If clinically stable, BP once a shift
  - *Less than 27 weeks* – If clinically stable, BP q6 or as indicated by infant status

No routine weights

- Use the Birth weight (BW) unless otherwise indicated by clinical status
- If weight is required – MUST be done as a 2 person process

Pain Management and Comfort

- Minimize noise in the room
- Monitor PIPP scores regularly
- Provide **OIT (Oral Immune Therapy)** regularly as an option for additional comfort measures

Minimal Handling

- **No routine linen changes**: If required – MUST be done as a 2 person process
- Removal of polyethylene occlusive plastic wrap as a 2 person process
- **No abdominal girth** unless clinically indicated
- **No bathing** unless clinically indicated (Infant born to mother with HIV)
- **No axilla temperatures** – use skin probe temperature – unless clinically indicated for correlations
- **Head Circumference** (HC) to be performed in Resuscitation Room – avoid lifting head
- **Assessments** (healthcare team) must be kept to a minimum
    - Do not exceed 6 h without a full assessment. Group all assessments
    - ALL examinations should be focused and brief
    - Nursing to make every effort to collaborate with medial team for a group assessment
- **Diaper care -** lift from the hips and slide under (do not lift legs). Leave diaper open if possible for ELBW
- **Fontanelle checks** – No routine assessments – may be deferred for 72 h (unless clinically indicated)

Skin to Skin

- Make every attempt to keep head and body midline (consider side-lying)
- Infant transfer MUST be a 2 person process
- Encourage "Hand Hugging" if unable to do Skin to Skin

Tests and Procedures

- **Arterial line sampling** – technique – slow and steady
- Continue with glucose testing (glucometer) as per policy or as ordered by MD/NP
- Glucose by the UAL if present (no heel prick if UA). If correlating heel prick is required – contact medical team for approval
- Lumbar puncture should be avoided until after 72 h
- If infant requires an invasive procedure (PAL, chest tube, PICC line) consider Fentanyl bolus prior to procedure
- **Head Ultrasound** – will require 2 person to adjust positioning as required to complete the test (US machine may need to enter room on opposite side if infant is in OMNI bed and backwards)
- **Targeted ECHO** should be done at 24 or 72 h (less than 15 min). No routine ECHO unless otherwise clinically indicated
- Routine **head ultrasounds** should be deferred until 72 h unless there are specific decision making concerns
- Routine swabs may be deferred for 72 h – contact Infection Control to confirm reason for ordered test
- **Research and Studies -** Permitted as approved by Staff MD

Transfer and X-Ray

- Keep the OMNI bed for all babies less than $26^{6/7}$ ELBW for 72 h
- **X-ray** - avoid levelling the bed if possible – consider dorsal-decubitus (cross-table) view
